# Selective industry adoption of a voluntary front-of-pack nutrition label results in low and skewed uptake: 10-year results for the Health Star Rating

**DOI:** 10.1038/s41430-024-01480-2

**Published:** 2024-08-06

**Authors:** Mariel Keaney, Damian Maganja, Eden Barrett, Simone Pettigrew, Alexandra Jones

**Affiliations:** 1 The George Institute for Global Health, UNSW, Sydney, NSW Australia; 2 School of Health Sciences, Faculty of Medicine and Health UNSW, Sydney, NSW Australia

**Keywords:** Health policy, Nutrition

## Abstract

Front-of-pack nutrition labelling (FOPNL) on packaged foods is recommended by the World Health Organization to promote healthier diets. Our aim was to assess uptake of Australia’s FOPNL, the Health Star Rating (HSR), overall and by HSR score received (0.5 (less healthy) to 5.0 (more healthy) in 0.5 increments) after ten years of voluntary implementation. In a sample of 21,197 products, we found HSR uptake of 37% overall in 2023. Uptake was unevenly distributed, with 24% of products with an HSR ≤ 3 displaying the label, compared to 53% of products scoring ≥3.5 (*p* < 0.001). Low HSR uptake on poorly rated products demonstrates that most food manufacturers will only display FOPNL where marketing value exists in a voluntary system. Poor and uneven HSR uptake limits consumers’ ability to meaningfully use the label to compare and choose healthier products. Government action is urgently required to make the HSR system mandatory.

## Introduction

Unhealthy diets are a leading cause of chronic disease in Australia and globally [1, 2]. The World Health Organization recommends front-of-pack nutrition labelling (FOPNL) on packaged foods as an evidence-based, cost-effective ‘best buy’ policy to promote healthier diets and prevent noncommunicable diseases [3]. Simple, graphical FOPNL can assist consumers to compare the nutritional quality of foods to support healthier choices and encourage food manufacturers to reformulate their products to make them healthier.

Australia and New Zealand use the Health Star Rating (HSR) FOPNL system, whereby a summary of the healthiness of a product is displayed as a rating ranging from 0.5 stars (least healthy) to 5.0 stars (most healthy) in half-star increments. Introduced as a government-led but voluntary scheme in 2014, relatively low uptake of the HSR system has been observed overall, with the label displayed on approximately one-third of products in 2023 [4].

In 2019, targets for voluntary uptake of the HSR system were established by Australian and New Zealand governments, initially aiming for 50% of ‘intended products’ to display the label by November 2023, with mandating the HSR system to be considered if uptake does not reach 70% by November 2025 [5]. This study aimed to build upon recent reporting of overall low HSR uptake by examining the distribution of uptake by HSR increment of products in Australia [4].

## Methods

The FoodSwitch database contains labelling and nutrition composition data for packaged supermarket foods, with data systematically collected and cleaned annually by trained teams [6]. For this paper, we used data from four large Australian supermarket retailers collected between March and June 2023.

Recorded products are classified into a hierarchical category tree [7], which were used to determine which products were not an intended product within the HSR system, in accordance with existing government guidance [5]. Of 22,147 intended products identified within the FoodSwitch 2023 database, an HSR could not be assigned for 230 products (primarily variety packs and meal kits) that displayed multiple HSRs or for which required information was missing. The remaining 21,917 products were included in the analysis.

HSRs for intended products were obtained from either the HSR displayed on pack or, for products that did not display an HSR (including those still carrying the now defunct energy icon only variant [8]), an HSR calculated from other information provided on pack.

We calculated HSR uptake by dividing the number of products displaying the HSR label by the total number of intended products, both overall and by each HSR increment. To examine whether HSR label uptake differed by ‘healthiness’, we compared uptake of products scoring HSR ≥ 3.5 and products scoring HSR ≤ 3 using Pearson’s chi-square test. The mean HSR of products displaying the HSR label was compared against those not displaying the label using Welch’s *t*-test. Analyses were conducted in Stata/BE version 18.0.

## Results

**Fig. 1:**
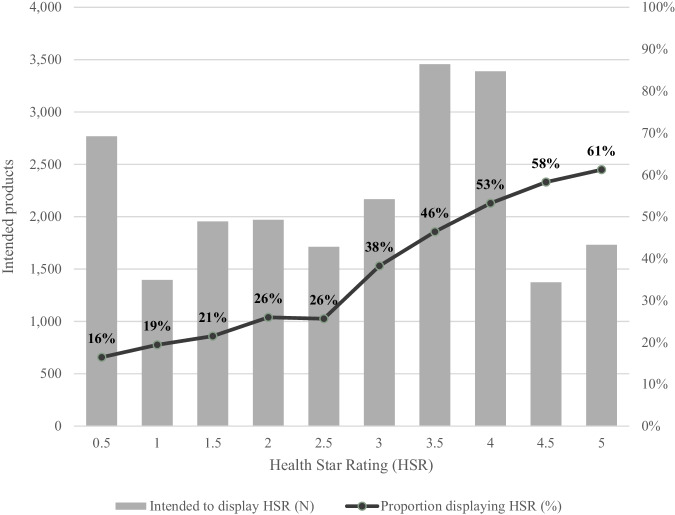
Uptake (%) of the Health Star Rating (HSR) label by star rating in 2023.

Of 21,197 intended products, 8193 (37%) displayed an HSR. Uptake was higher amongst products with higher ratings. The lowest HSR uptake was observed in products with an HSR of 0.5 (16% of products displaying), while the highest uptake was observed for products with an HSR of 5.0 (61%) (Fig. 1). Only 24% of products with an HSR ≤ 3 displayed the label, compared to 53% of products scoring ≥3.5 (*p* < 0.001). The mean HSR of products displaying an HSR was 3.4, compared to 2.4 for products not displaying an HSR (*p* < 0.001).

## Discussion

We found differential uptake of the HSR system by rating, with higher (i.e. more favourable) ratings more likely to be displayed. This indicates reluctance to display a low HSR. Such skewed uptake of the HSR system undermines its potential as a public health tool.

Our findings on uneven uptake are consistent with previous studies. Similar studies using 2017 and 2019 data found that higher-scoring products were more likely to display the HSR label [9, 10], and recent reporting by product category showed that uptake ranged widely from 5% in low-scoring categories to 90% in high-scoring categories [4]. Our new analysis suggests this inconsistent uptake persists.

Our results show that the HSR system in its voluntary format is continuing to be used as a marketing tool by the food industry where products score well. Selectively displaying the HSR limits its utility to consumers as a public health intervention if they are unable to meaningfully compare products and have limited ability to identify and avoid unhealthy foods. With nearly a decade since the introduction of the HSR system and continued plateauing of uptake overall, this selective application reinforces the need for Food Ministers to commence transitioning to a mandatory scheme now to achieve HSR’s public health potential without further delay [4].

This analysis contributes to findings from previous year on year uptake trends using a comprehensive dataset [9]. Strengths include the use of a comprehensive, systematic collection of products available in Australian supermarkets. By examining uptake by HSR increment, our findings add to governmental reporting which only provides overall uptake. Limitations include the HSR calculation for products not displaying an HSR label, which was estimated using ingredient lists, food composition databases, and other sources particularly for products where fruit, vegetable, nut and legume content, and fibre information was not listed on pack [6].

## Conclusion

These findings provide further evidence that immediate action to mandate the HSR system is required to provide consumers with this easy-to-use summary information on all products. A mandatory system will also provide a level playing field for industry and better incentivise product reformulation to achieve higher ratings when HSR information can no longer be withheld from consumers. Ongoing monitoring of HSR uptake overall and by rating is necessary to hold both the food industry and government to account for ensuring FOPNL information is provided to consumers.

## References

[1] Crosland P, Ananthapavan J, Davison J, Lambert M, Carter R. The health burden of preventable disease in Australia: a systematic review. Aust N Z J Public Health. 2019;43:163–70. 30830711 10.1111/1753-6405.12882

[2] Afshin A, Sur PJ, Fay KA, Cornaby L, Ferrara G, Salama JS, et al. Health effects of dietary risks in 195 countries, 1990–2017: a systematic analysis for the Global Burden of Disease Study 2017. Lancet. 2019;393:1958–72. 30954305 10.1016/S0140-6736(19)30041-8 PMC6899507

[3] World Health Organization. Tackling NCDs: best buys and other recommended interventions for the prevention and control of noncommunicable diseases, 2nd ed. Geneva: WHO; 2024. Report No.: CC BY-NC-SA 3.0 IGO.

[4] Laznik N, Dunford E, Jones A, Howes K, Taylor F. FoodSwitch-State of the Food Supply. A Five-Year Review. Australia: The George Institute for Global Health; 2023.

[5] Department of Health. Uptake of the Health Star Rating system as at November 2023. 2024. Available from: http://www.healthstarrating.gov.au/internet/healthstarrating/publishing.nsf/Content/target-and-intended-products.

[6] Dunford E, Trevena H, Goodsell C, Ng KH, Webster J, Millis A, et al. FoodSwitch: A Mobile Phone App to Enable Consumers to Make Healthier Food Choices and Crowdsourcing of National Food Composition Data. JMIR MHealth UHealth. 2014;2:e3230. 10.2196/mhealth.3230 PMC4147708 25147135

[7] Dunford E, Webster J, Metzler AB, Czernichow S, Mhurchu CN, Wolmarans P, et al. International collaborative project to compare and monitor the nutritional composition of processed foods. Eur J Prev Cardiol. 2012;19:1326–32. 21971487 10.1177/1741826711425777

[8] Department of Health. Health Star Rating system: Post Five-Year Review Monitoring Framework. 2023. Available from: http://www.healthstarrating.gov.au/internet/healthstarrating/publishing.nsf/Content/01C15064FB52327BCA25861D00364E60/$File/Monitoring%20Framework%20-%20final.PDF.

[9] Shahid M, Neal B, Jones A. Uptake of Australia’s Health Star Rating System 2014–2019. Nutrients. 2020;12:1791. 32560224 10.3390/nu12061791 PMC7353262

[10] Jones A, Shahid M, Neal B. Uptake of Australia’s Health Star Rating System. Nutrients. 2018;10:997. 30061512 10.3390/nu10080997 PMC6115967

